# The Prevalence of Generalised Anxiety Disorder Among Prisoners of the Penitentiary Institution in North-Eastern Poland

**DOI:** 10.3389/fpsyt.2021.671019

**Published:** 2021-06-14

**Authors:** Barbara Stawinska-Witoszynska, Katarzyna Czechowska, Waclaw Moryson, Barbara Wieckowska

**Affiliations:** ^1^Department of Epidemiology and Hygiene, Chair of Social Medicine, Poznan University of Medical Sciences, Poznan, Poland; ^2^Department of Computer Science and Statistics, Poznan University of Medical Sciences, Poznan, Poland

**Keywords:** generalised anxiety disorder, mental health, prisoners, penitentiary system, prevalence

## Abstract

**Introduction:** Generalised anxiety disorder (GAD) is one of the most common mental disorders. The psychosocial factors that may lead to generalised anxiety disorders include stress, traumatic events, conscious and unconscious internal conflicts, and low social and economic status. Imprisonment and forced isolation may favour the development of depression or anxiety disorders in inmates. Thus, this study aimed to analyse the prevalence of generalised anxiety disorder (GAD) in the population of inmates detained in one of the largest penitentiary units in north-eastern Poland.

**Materials and Methods:** The data comes from 2017 and includes information on 635 male inmates incarcerated at the Czerwony Bór Prison. The information comes from the health records of individual inmates, kept by the prison outpatient clinician, and documented consultations with doctors of units outside the prison. The classification of generalised anxiety disorders (F41.1) was made in accordance with the 10th Revision of the International Statistical Classification of Diseases and Health Problems and the clinical diagnosis made by a psychiatrist. The Mann-Whitney test was used to compare quantitative data without normal distribution, chi-squared test or Fisher exact test for comparing qualitative data. A one-dimensional and multi-dimensional logistic regression model was used to examine the impact of the prison type on the prevalence of generalised anxiety disorder.

**Results:** Generalised anxiety disorder was observed among 44 prisoners (6.9%), most often in the age range 30–39 years and among men younger than 30 years, respectively 40.9 and 31.8% of all diagnosed. The average age of patients was 34.6 years. The least number of prisoners with GAD was in the age group 50–59 (2.3%). Nearly 66% of patients were prisoners detained in a closed type prison; the chance of generalised anxiety disorder was three times higher than among the prisoners in a half-open and open type facility. Generalised anxiety disorder was diagnosed significantly more often with those currently serving a prison sentence than those before incarceration.

**Conclusions:** In Polish conditions, the importance of the problem associated with GAD is evidenced by a significant increase in its prevalence in the prison environment and a three times higher chance of developing generalised anxiety disorder among prisoners in a closed type institution, which calls for highly organised psychiatric care and increased availability of psychological assistance for prisoners.

## Introduction

Generalised anxiety disorder (GAD) is one of the most common psychiatric disorders (1). Although GAD runs a less visible course than schizophrenia, depression or bipolar disorder, it may similarly result in the patient's disability (2, 3). Generalised anxiety disorder is characterised by the chronicity of the process (lasting more than 6 months), intense feelings of anxiety and persistent yet ungrounded worrying about various problems (e.g., money, future, family or health) (4–7). Other symptoms that patients experience include headaches and dizziness, restlessness, easy fatigue, sleeping problems, tremors, tachycardia, dyspnoea, palpitations, gastrointestinal complaints, e.g., difficulty swallowing, epigastric pain, and excessive perspiration (1, 3–8).

It should be noted that the GAD (generalised anxiety disorder) problem is generally marginalised. There are difficulties in diagnosing this syndrome from other mental disorders, especially when GAD and depression coincide. The efforts of psychiatric neuroimaging, genetic and neurochemical studies are focused on finding biomarkers that play an essential role in its aetiology and treatment (9).

Among other psychosocial factors, stress, traumatic events, adverse events experienced in childhood and more recently before the onset of symptoms, conscious and unconscious internal conflicts, and low social and economic status may lead to generalised anxiety disorder (10–12).

The more frequent prevalence of mental disorders, including anxiety disorders observed among immigrants, is explained by discrimination, yet according to many researchers, other factors such as economic stress or lack of life partner are of substantial significance (13–15). It is difficult to determine the dominant cause of the disorder. In the group of Hispanics working on daily payment, people with low social status, the prevalence of depression and anxiety turned out to be higher than in the general population of Latinos in the U.S., on the other hand, among Puerto Rican youth living in the Bronx, anxiety disorders were more common than those in Puerto Rico, at a similar level of poverty in both groups (14, 15). Cigarette smoking or quitting, alcohol abuse, and taking drugs include other risk factors for generalised anxiety disorder (16–18). The relationship between smoking and anxiety disorders has not been confirmed by studies (17).

Undoubtedly, deprivation of liberty and the nature of life in prison can be regarded as stress factors. Apart from masculinisation and predominance of young men, prisoners constitute a distinctive group differing in many respects from other groups in the society (14, 17, 18). They are often individuals from pathological backgrounds, with low social and economic status, suffering from illnesses that frequently result from inappropriate lifestyles. There is a risk that prisoners may be particularly vulnerable to the emergence of mental disorders or exacerbating the existing mental ones. It can be influenced by their background and the difficult conditions of confinement, such as overcrowded prisons, isolation from the world outside, the need to adapt to the internal order within the prison, violence, and a lack of support. (4, 5, 9, 13, 14, 19–21).

In Poland, convicts are classified according to such factors as gender, age, previous imprisonment, time remaining until completion of the imprisonment, type of offence and act committed (intentional or unintentional), their state of health, degree of demoralisation and the risk they pose to the society. Prisons are organised as closed prisons, semi-open prisons and open prisons. These three types differ in the degree of security they provide and the type of isolation the prisoners undergo (22).

As incarceration evokes mainly negative emotions, which favours the development of depression or anxiety disorders in inmates, this study aimed to analyse the prevalence of generalised anxiety syndrome in inmates detained in one of the largest penitentiary units in north-eastern Poland.

## Materials and Methods

In the descriptive epidemiological study, only secondary sources of information were used - medical records of 635 prisoners aged 21 to 72 from the Penal Institution in Czerwony Bór, located in the Podlasie Voivodship, containing adult men, convicted for the first time and recidivists. It is a penitentiary unit, divided into three types of establishments: closed, semi-open and open, differing in the way of securing residential buildings and the duties of officers serving in a given category of the prison. The data for this study was obtained from the prisoners' medical records dating back to the period before incarceration and their health records provided by the prison physician supplemented with the results of specialist consultations from the medical units outside the prison and the results of additional diagnostic tests. Generalised Anxiety Disorder (F41.1) was classified in accordance with the 10th Revision of the International Classification of Diseases and Health Problems (23). The authors chose to discuss GAD in detail because its prevalence was higher than that of alcohol dependence syndrome and depression. Moreover, the reliability of the diagnosis was higher than that of personality disorders that are difficult to diagnose and for which, depending on the classification of the diseases adopted, minor differences in typology are encountered. The following variables were included in the analysis: age of prisoners (5 age groups - under 30, 30–39, 40–49, 50–59, 60 years and more), the type of prison (closed, semi-open including open due to a small number of convicts in an open prison), classification of prisoners (first-time offender or recidivist), length of previous sentence and occupation.

The time of diagnosis was determined (before or during imprisonment), which allowed estimating the number of newly diagnosed cases of generalised anxiety disorder during detention. A psychiatrist made the diagnosis regardless of whether the clinical diagnosis had been made before admission to the Penitentiary Institution or at the time of incarceration.

### Statistical Analysis

The general characteristics of the study group were given in numbers and percentages by individual category. The Mann-Whitney test was used to compare quantitative data without normal distribution, chi-squared test (or Fisher exact test, when the numbers of individual categories were too small) for comparing qualitative data. A one-dimensional and multi-dimensional logistic regression model was used to examine the impact of the prison type on the prevalence of generalised anxiety disorder. Due to the relatively small patient group in the multi-dimensional model, the adjustments were made in stages. First, only the variables significant in the unidimensional analysis were adjusted, then all the collected variables. To estimate the detection of disease among prisoners before and during imprisonment, a one-sample test was used. Testing it was assumed, that no difference in proportions means the same, i.e., 50%, frequency of diagnosing diseases before and during detention in prison.

In statistical analyses, the PQStat v1.6.4 program was used, and the significance threshold was set at 0.05.

## Results

The surveyed population consisted predominantly of young males aged 30–39 and those under the age of 30. Most of them had been first-time offenders, serving 1 to 5 years in prison. In general, the length of sentences adjudicated ranged from one to 25 years of imprisonment. A total of 255 prisoners (40.2%) were placed in a closed-type institution. Almost 50% of the prisoners were working. Anxiety disorders were diagnosed in 6.9% of the prisoners and depression in 1.7% (Table 1).

**Table 1:** General characteristics of the study population.

**Variables**	**Count = 635 (100%)**
**Type of penitentiary**	
Closed	255 (40.2%)
Open and semi-open	380 (59.8%)
**Length of adjudicated sentence served**	
Up to 1 year	206 (32.4%)
1–5 years	349 (55.0%)
5 years or more	80 (12.6%)
**Classification**	
First-time incarceration	377 (59.4%)
recidivist	258 (40.6%)
**Work situation**	
Working	322 (50.7%)
Not working	313 (49.3%)
**Age**	
Under 30	205 (32.3%)
30–39	227 (35.7%)
40–49	110 (17.3%)
50–59	63 (9.9%)
Over 60	30 (4.7%)
**Selected mental disorders**	
Anxiety disorders	44 (6.9%)
Depression	11 (1.7%)

Generalised anxiety disorder occurred in 6.9% of respondents, mainly in younger age groups. Most patients were observed in the age range 30–39 years below 30 years, respectively, 40.9 and 31.8% of all convicts with generalised anxiety syndrome. Among 50–59. only one case of this disorder was found (Table 2).

**Table 2:** Assessment of the prevalence of generalised anxiety disorders according to the age of prisoners, their classification, type of prison, length of imprisonment to date, work activity, time of diagnosis and depression.

	**Generalised anxiety disorder**		
**Variables**	**Yes (*n* = 44)**	**No (*n* = 591)**	***p*-value**
Type of penitentiary			0.0003
Closed	29 (65.9%)	226 (38.2%)	
Open and semi-open	15 (34.1%)	365 (61.8%)	
Length of adjudicated sentence served			0.2097
Up to 1 year	11 (25%)	195 (33%)	
1–5 years	24 (54.5%)	325 (55%)	
5 years or more	11 (25%)	195 (33%)	
Classification			0.7802
First-time incarceration	27 (61.4%)	350 (59.2%)	
recidivist	17 (38.6%)	241 (40.8%)	
Work situation			0.4699
Working	20 (45.5%)	302 (51.1%)	
Not working	24 (54.5%)	289 (48.9%)	
Age median (25–75%)	32.5 (26–42)	34 (28–43)	0.3697
Below 30	14 (31.8%)	191 (32.3%)	
30–39	18 (40.9%)	209 (35.4%)	
40–49	9 (20.5%)	101 (17.1%)	
50–59	1 (2.3%)	62 (10.5%)	
Over 60	2 (4.6%)	28 (4.7%)	
Depression			0.0346
Yes	3 (6.8%)	8 (1.4%)	
No	41(93.2%)	583 (98.6%)	

There was a significant difference between the occurrence of generalised anxiety disorder and the type of prison (*p* = 0.0003). Among the inmates with generalised anxiety disorder, depression was observed significantly more frequently compared to the inmates who did not suffer from this disorder (*p* = 0.0346). There was no relationship between the age of the inmates, the length of the sentence served to date, the classification of the inmates (first-time offender, recidivist), their work situation and the prevalence of generalised anxiety disorder (Table 2).

The risk of generalised anxiety disorder, as determined by the unadjusted model, was ~3 times higher for prisoners serving their sentence in a closed-type institution OR (95% CI) = 3.1 (1.6; 6.0) (Table 3). A statistically significant odds ratio was also obtained in the other two regression models, the minimally adjusted model as well as the full model. Depression and other analysed variables (length of sentence served to date, classification, work situation and age) had no significant effect on the relation between prison type and the occurrence of generalised anxiety disorder (Table 3).

**Table 3:** Regression models describing the effect of incarceration on the prevalence of generalised anxiety disorder in prisoners.

	**Logistic regression model**		
	**One-dimensional (without adjustment)**	**Minimally adjusted**	**Fully adjusted**
	**OR [95%CI]**	AOR^*^ **[95%CI]**	**AOR**^**#**^ **[95%CI]**
Generalised anxiety disorder	3.1 [1.6; 6.0] *p* = 0.0005	3.1 [1.6; 5.9] *p* = 0.0007	3.0 [1.5; 5.8] *p* = 0.0017

*OR [95%CI], anxiety disorders odds ratio*.

*AOR [95%CI], adjusted odds ratio*.

* *Adjusted for depression*.

# *Adjusted for depression, length of imprisonment served to date, classification, work situation and age*.

Table 4 presents the percentage of diagnoses of generalised anxiety disorder in the studied population depending on the time of the diagnosis. These disorders were diagnosed more often among prisoners while staying in prison (86.4%). A significant difference was found between the number of diagnoses made before and during the incarceration (*p* < 0.0001).

**Table 4:** The prevalence of generalised anxiety disorder in the population of prisoners depending on the date of diagnosis.

**Diagnosis of generalised anxiety disorder** ***n*** **=** **44**	
Before arriving at the prison	6 (13.6%)
During imprisonment	38 (86.4%)
*p*-value	<0.0001

*N, number of prisoners*.

## Discussion

The prevalence of all anxiety disorders is estimated differently, depending on the country and research methodology, in the range from a few to several per cent (1, 2). Generalised anxiety disorder (generalised anxiety syndrome) making up the group of mental disorders is one of the most commonly detected mental diseases (3), and although they are less visible than schizophrenia, depression or bipolar disorder can also lead to patient disability (1, 6).

During the entire lifetime, generalised anxiety disorder occurs in the U.S. population at the level of 5.1 to 11.9%; in Europe, its prevalence is lower 4.3–5.9%, on average 5% (10, 24, 25).

The consistency of most research results concerns the more frequent prevalence of generalised anxiety disorder (GAD) among women and adolescents as well as in younger adults than in older age (11, 24, 26, 27).

In studies conducted in Poland, generalised anxiety disorder occurred in 6.9% of the prisoners' population and was one of the most frequently diagnosed disorders. Identical to the results of most epidemiological studies, they mainly concerned younger age groups. Most diagnosed were observed among 30–39-year-olds and prisoners under 30 years old, while in the two oldest age ranges, the cases of generalised anxiety disorder occurred sporadically. The prevalence of anxiety disorders among prisoners was much higher than that of the general Polish population. Based on the EZOP Polska study, the first cross-sectional epidemiological study in the country using the structured diagnostic questionnaire CIDI, conducted on a sample representative of the population aged 18–64 years, generalised anxiety disorder during life was found in 1.1% (95% CI 0.9–1.3), significantly more often among women (1.5%) than men (0.6%) (28). Unlike other studies, which may be the result of their diverse methodology, they were the least frequent among the youngest respondents, regardless of gender (11, 24, 26–28).

In similar masculinised (90% men) and young prisoners population in the north-east of Amhar in Ethiopia with an average age of 30.6 ± 11.49 SD, the prevalence of anxiety disorder was generally 36.1% and was higher compared to other low- and middle-income countries such as Chile or India. In European prisons, the prevalence of depression or anxiety disorders is estimated at around 25% (16).

Compared to the results of the above studies, the low prevalence of generalised anxiety disorder among the prison population studied may be puzzling. This phenomenon might have been influenced by the type of the study, which was the only one that was granted consent, and with the exclusive use of data from prisoners' medical records. The analysis took into account all diagnoses of generalised anxiety syndrome made by a consultant psychiatrist working at the prison outpatient clinic and the previous diagnoses made by the inmates' psychiatrists before incarceration and verified by the consultant. Generalised anxiety syndrome had been diagnosed prior to detention only in 13.6% of the surveyed, which makes it impossible to associate it with the fact of imprisonment. The cause of its occurrence could not be established. Unfortunately, the prisoners' medical records were sometimes incomplete, and some of them had not received any medical care before their detainment. All prisoners treated at the outpatient clinic due to mental problems or referred by the psychologists working with prisoners were offered psychiatric consultations. As a result, the number of those diagnosed with generalised anxiety disorder increased.

We also recognise that the group of inmates with generalised anxiety syndrome was relatively small (44 inmates with the syndrome and 591 with no disorder). Logistic regression is sensitive to small groups, especially when models contain many adjustment variables. Although stable results of the full model were obtained, which confirmed the results obtained in smaller models, it would be worthwhile to extend such a study to a larger population of detainees.

On the other hand, the prevalence of generalised anxiety disorders among prisoners appeared to be much higher than in the general population of Poland, where the prevalence of this phenomenon was at a low level, and cross-sectional epidemiological studies had been conducted on a representative group of respondents. The surveyed prisoners from the penitentiary in Czerwony Bór originated from the general Polish population.

Despite its limitations, the opportunity to carry out an epidemiological study in the population of prisoners in the penal facility in Czerwony Bór made it possible to determine the level of prevalence of generalised anxiety disorder depending on various variables and to select those inmates particularly at risk of this disorder. As studies on the health of the prison population carried out by doctors in Poland are scarce, this paper may evoke the interest of researchers who might want to broaden their knowledge on the subject.

Unfortunately, the insufficient number of studies regarding prisoners, especially in our part of Europe, makes it difficult to compare the results of this study with other findings.

Among Ethiopian prisoners, the risk of developing anxiety disorders was 2.49 times higher in those who described their lives before imprisonment as “unhappy” (16). Probably, in this case, the existing anxiety symptoms have been superimposed on embedding stress. Polish prisoners during control visits to the prison dispensary often reported lowered mood, sleep problems, headaches and dizziness, shortness of breath, and increased anxiety. Their stay in prison resulted in the prevalence of generalised anxiety disorder, which was diagnosed in most prisoners during their imprisonment, as already mentioned, similar to American studies (18). The study showed that the chance of developing generalised anxiety disorder among closed-type prisoners was more than three times higher than among prisoners in semi-open and open facilities, regardless of the age of the prisoner. Depending on the severity of the crime and the length of the sentence, three types of prisons operate in France. The results of studies conducted among French convicts, with a median age of 37 years, which were given with an error of fraction estimation, showed the prevalence of clinically significant mental disorders estimated based on the agreement of two clinicians in 27.4 ± 4.5% of prisoners. The prevalence of generalised anxiety disorder itself, diagnosed according to two clinicians, was estimated at 12.0 ± 42.1% and diagnosed by at least one - at 19.6 ± 3% (20). Among the respondents, almost half (49%) men were in prison at least for the second time, 28% reported child abuse, 29% imprisonment of a family member, 16% were psychiatrically treated (20). The authors of this publication drew attention to the difficulties in interpreting psychiatric diagnoses in prisons, especially using traditional standardised interviews (20). They also noted that depression was more likely to occur in recidivists, while anxiety disorders were more common among first-time offenders (20). In our study, there was no association between the length of sentence served to date and the classification of prisoners (first-time offender, recidivist) and the prevalence of generalised anxiety disorders.

Attention is paid to the issue of the importance of determining the level of psychological stress of prisoners placed in prisons, thanks to which it is possible to predict what health problems they may have, depending on the conditions prevailing in individual prisons (29). The scale of the problem was confirmed, among others, by studies in Italy, Spain and Nigeria, according to which prisoners, despite health problems, are not properly treated (30–32). Lack of therapy for convicts with mental disorders may lead to deterioration of the quality of life of prisoners and suicide attempts, also described in general populations of different countries (20, 33, 34). In psychiatric disorders, including anxiety disorders, effective and integrated therapy is required, which includes both pharmacotherapy, psychotherapy and behavioural therapy (35–37). For obvious reasons, in prison conditions, it is extremely difficult to achieve sufficient conditions to achieve satisfactory results, for example, due to restrictions on the freedom of changing persons' place of stay and uninhibited undertaking physical activity that can have a positive effect on treatment (38). Worldwide, there is a greater need for the treatment of persons deprived of their liberty and for the undertaking therapy of mental illnesses, compared to the general populations. In the case of anxiety disorders, this problem concerns 17.2–58.6% of prisoners (39–41).

Summarising, the prevalence of generalised anxiety disorder in the male population in one of the largest prisons in north-eastern Poland was definitely higher compared to the general population of Poland but lower than among prisoners in many other countries. In Polish conditions, the importance of the problem associated with GAD is evidenced by a significant increase in its prevalence among prisoners. There is three times greater chance of developing generalised anxiety disorder among prisoners in a closed type institution, which requires a better organisation of psychiatric care and increased availability of psychological support offered to prisoners.

## Data Availability Statement

The raw data supporting the conclusions of this article will be made available by the authors, without undue reservation.

## Ethics Statement

The studies involving human participants were reviewed and approved by Ethics Committee of the Poznan University of Medical Sciences, Collegium Maius, Fredry street 10, 61-701, Poznań. Written informed consent for participation was not required for this study in accordance with the national legislation and the institutional requirements.

## Author Contributions

BS-W and KC: conceptualization, investigation, resources, and data curation. B-SW, KC, and BW: methodology. BW: software and formal analysis. B-SW: validation, supervision, and project administration. B-SW, KC, and WM: writing—original draft preparation, writing—review and editing, and visualization. All authors contributed to the article and approved the submitted version.

## Conflict of Interest

The authors declare that the research was conducted in the absence of any commercial or financial relationships that could be construed as a potential conflict of interest.
